# Supplementary material for: Online therapy with families ‐ what can families tell us about how to do this well? A qualitative study assessing families’ experience of remote Dyadic Developmental Psychotherapy compared to face-to-face therapy
Source: PLoS One. 2024 Apr 16;19(4):e0301640. doi: 10.1371/journal.pone.0301640 (PMC11020366; doi:10.1371/journal.pone.0301640)
Supplement: S1 File — (DOCX) [file pone.0301640.s001.docx]

# **Interview excerpts**

“it's really quiet up there, she's in her own environment...her comfortable area, her bedroom is very much the way she likes it... I should imagine it helps massively, typically when you're talking about things that are tricky, anxiety, anger, you know.” – Hannah (Interview 2)

“So, leaving my side, going to therapy or being involved with the study, outside of the home environment would have been difficult for him...the virtual meetings are working perfectly, it’s convenient.” – Jill (Interview 1)

“Isaac did seem to really open-up and kind of started to talk about something that happened when he was a baby…I think it really was about being in the room at that point...there was a cake in the reception... and it was actually whilst he was eating that, that it happened... I think it was kind of a particular set of circumstances.” – Stephen and Greg (Interview 6)

“Yeah, I think when he’s in a room, when he went into the room with the therapist  and they would be interacting, you know, drawing, that really sucked him in… he was sitting next to her [the therapist] on the floor talking to her.” – Susan (Interview 5)

“I think, because young people of her age are so comfortable with screens, I think it's almost easier to say stuff and share stuff.” – Hannah (Interview 2)

“I don’t think he would have engaged as much. I think the screen is a distraction, especially with other faces, it’s a big distraction, he’s very easily distracted, so I don’t think that would have worked very well.” – Susan (Interview 5)

“I think he would not have been able to have taken it as seriously, I think it would have been just going through the motions, just sort of attending and saying what he thought people wanted to hear. I don’t think we would be where we are now, we wouldn’t be as far as we are, I think it would have taken a lot longer.” – Anne (Interview 3)

“His concentration level is very poor, but for some reason...the whole on-line learning and zoom calls and so-forth throughout the beginning of the pandemic worked so well for him surprisingly, because he was able to just sit there and concentrate and that was not him [pre-covid], I found it incredibly satisfying.” – Jill (Interview 1)

“but one of the real positive things was on the screen, especially from the physical contact because it’s one of Jason’s things he doesn’t necessarily like, especially if he’s trying to isolate, he will put himself at the other side of the room... One of the things we realise…was having the screen automatically put him next to you.” – Henry (Interview 4)

“I think maybe there is something in the idea that we go to this special place to do this thing, and then we go away again…there is a very clear boundary which may well be a good thing.” – Stephen and Greg (Interview 6)

“The trouble of being home is right at the end you’re walking from your front room into the kitchen, and you haven’t got any decompression.” – Henry (Interview 4)

“It’s just helpful to compartmentalise it, you know.” – Stephen and Greg (Interview 6)

“Definitely for Jason the driving back was decompression, as I said, quite often he would literally just go to sleep for the twenty minutes, half an hour.” – Henry (Interview 4)

“So, I think at home it’s that…we didn’t see it straightaway, but if we didn’t engage with him [following the DDP session] we quite often saw more challenging behaviour after the remote sessions if we didn’t manage the post-session element.” – Henry (Interview 4)

“In the beginning with the thought of the travel and getting there and it was the paramount thing... it was hard work to push to have to go. Seeing the relationship change…that tells me that it’s worth it.” – Anne (Interview 3)

“In terms of travel, so if it was the choice between doing this with a two-hour journey, or doing it at home, I think practically we’d have to do it at home.” – Stephen and Greg (Interview 6)

“I'd have travelled to outer Mongolia to try and get Megan the help and get us the support that we needed...we would have done it, but it would have added to the stress of what was already a very stressful situation.” – Hannah (Interview 2)

“You miss the personal connection, you know, the interaction. I think that’s one of the main downsides of on-line, virtual meetings, it’s not so easy to read people’s body-language.” – Jill (Interview 1)

“I think Adam [DDP therapist] is also able to see how Isaac’s reacting to things much more easily. I think also the difficulty of being on-line is that Isaac has to be in one place for it to work, whereas in the room Isaac can actually wander around a bit, play with this, look at that.” – Stephen and Greg (Interview 6)

“for our children you are almost cutting off half the body, you are cutting off half of their communication…So, where you are cutting half that body language off for them, it’s almost as if you’re covering an ear or covering an eye. I think, it does put them at a bit of a disadvantage.” – Anne (Interview 3)

“I think, as far as looking at Adam is concerned, and seeing how he's responding to what we're saying, that's absolutely fine, because we see him...the way we set screens up, we can see, most of him, and we would be the same as if we were there in person” – Hannah (Interview 2)

“I think it’s down to the person [therapist], not just down to the child. It’s the person, the way the person on that screen is interacting with a child, because they can only see their face, so the smile, the nods, the still making that sort of eye contact.” – Jill (Interview 1)

“I think that Adam was conscious of the fact that he couldn’t observe the full body, so sometimes when he was facilitating the session… he would take a step or two back, and go over something and just check.” - Henry (Interview 4)

“we’d gone to that space and there was a degree of trust between me and Adam and Jason, so that you could have the harder conversations.” – Henry (Interview 4)

“ I think it’s come after two years of everybody having quite a lot of practice at doing things remotely…so I think it was fine to do it that way.” – Stephen and Greg (Interview 6)

“I’m usually fine, because we zoom all the time, it's something we've got used to isn't it really.” – Hannah (Interview 2)

“ because of COVID… we had to do all that home-schooling,  so she's quite comfortable, so the technology in itself isn't an issue.” – Hannah (Interview 2)

“Jason is a digital native, so he was very comfortable with that concept.” – Henry (Interview 4)

“I don’t know how you’d do it on-line...unless they are going to send us toys in the post, or, like he can use his own toys...I don’t know what form it would take if we were to do it on-line.” – Stephen and Greg (Interview 6)

“There is a barrier…I got used to talking openly with people, you know on the screen, but my son didn’t.  My son goes to primary school and, in the end, I ended up sending him in because I couldn’t home-school him.”  – Susan (Interview 5)

“I think if we hadn't had the experience, then I think it would be much harder, possibly, but I think because we're so used to it, because of COVID and everything else that we don't really think about it.” – Hannah (Interview 2)
