# Supplementary material for: Online therapy with families ‐ what can families tell us about how to do this well? A qualitative study assessing families’ experience of remote Dyadic Developmental Psychotherapy compared to face-to-face therapy
Source: PLoS One. 2024 Apr 16;19(4):e0301640. doi: 10.1371/journal.pone.0301640 (PMC11020366; doi:10.1371/journal.pone.0301640)
Supplement: Attachment — Submitted filename: Response to Reviewers.docx [file pone.0301640.s002.docx]

**Rebuttal Letter**

**Response to Academic Editor**

***We would like to thank the reviewers for taking the time to read our manuscript. We have edited the manuscript to incorporate the valuable feedback. We have paid particular attention to the comments regarding our analysis and use of IPA.***

1. Please ensure that your manuscript meets PLOS ONE's style requirements, including those for file naming.

*All files included in our submission have been named appropriately in line with PLOS ONE requirements.*

2. If you’ve not already done so, consider depositing your raw data in a repository to ensure your work is read, appreciated and cited by the largest possible audience.

*Due to the nature of our study we collected data in the form of transcripts from interviews with participants. We are unable to ethically deposit our raw data in a repository as participants did not give consent to the publication of interview transcripts in full.*

3. In your Data Availability statement, you have not specified where the minimal data set

underlying the results described in your manuscript can be found. PLOS defines a study's minimal data set as the underlying data used to reach the conclusions drawn in the manuscript and any additional data required to replicate the reported study findings in their entirety. All PLOS journals require that the minimal data set be made fully available.

*We have reviewed the PLOS one Data Availability guidance, in particular, the guidelines for qualitative data. The minimal underlying data set can be found within the manuscript as direct quotations from participant interviews. We unfortunately are unable to submit the raw transcripts in their entirety or additional excerpts as this would cross ethical boundaries and violate the agreement to which the participants consented.*

4. Please include your full ethics statement in the ‘Methods’ section of your manuscript file. In your statement, please include the full name of the IRB or ethics committee who approved or waived your study, as well as whether or not you obtained informed written or verbal consent.

*A full ethics statement is now included in the Methods section (page 8, lines 174-175).*

**Response to Reviewers’ comments**

**ABSTRACT**

1.The abstract is too long. It is suggested to cut the abstract and concentrate it to be more logical.

*Thank you for this comment, we have now consolidated the abstract to be precise and logical.*

**INTRODUCTION**

1. p.4 : more details are needed regarding the ethical concerns emerging regarding the negative impact of “tele-mental-health” (p. 4, li 75-76)

*We agree with this suggestion and specific details of prominent ethical concerns have now been explained in this paragraph (pages 3 and 4, lines 70-78).*

2. There is a lack of transition between the first major part of the introduction on the evaluation of online therapies, and the second part on the Dyadic Developmental Psychotherapy.

*Thank you for bringing this to our attention, we have expanded this section to establish a smoother transition (page 5, lines 100-105).*

3. Typo p. 5, li 103: “Rooted in attachment theory; the primary goal of DDP” the semicolon must be replaced by a comma”.

*We have addressed this typo and checked for other grammatical errors throughout the paper. Thank you for highlighting this.*

4. The goals and major therapeutic targets of the DDP are well described on the child's side, but we don't really understand what is being worked on with the adoptive parents. Viewing Table 1, I understand that the DDP includes sessions for the child and sessions for the parents? This must be clarified.

*This is an important question and we agree that this was not made clear in the paper. The parent’s role in the therapy sessions have now been explained in more detail, and we have clarified the aims of parent only sessions (page 6, lines 123-130).*

**METHODS**

1. I strongly recommend that authors follow the COREQ criteria (Tong et al. 2007); most of the information required by this checklist is already mentioned in the manuscript, but some is missing. This guide would provide greater precision and clarity.

Thank you for this recommendation which we have taken on board. We have consulted and referred to the COREQ criteria and added more information to the manuscript to enhance the clarity of out study methodology.

*Thank you for this recommendation. We have consulted the COREQ checklist and where possible, we have added additional information to our methods section in line with the criteria set out in COREQ. In particular we have added a ‘Research Design’ (page 7, lines 163 -175) and ‘Research team and reflexivity’ (page 10, lines 221 - 229) section. We hope these steps taken have improved the quality of the reporting of our study.*

2. Are DDP sessions protocolised? More precisely, is there an average number of sessions? This question is linked to an inclusion criterion: “families (…) who had received at least one session of DDP”. Justification for this inclusion criterion? And I’m not sure the parents can fully answer to a question concerning their experience of therapy with only one session.

*Thank you for raising this important question. The number of DDP sessions families receive are not protocolised but are guided by the child’s progress and development with regards to their attachment and security within the family. Parents will work with the therapist without the child at first and when ready, as judged by the therapist, the child will join sessions. We have clarified this in the introduction (page 6, lines 123-130). Inclusion criteria included families who had conducted at least one DDP session with their child and therefore will have had multiple parent-only sessions. We endeavored to include a range of participants of different family backgrounds, children's ages, and delivery methods. In line with this we accepted families with a range of experience of DDP including those with limited experience as this could provide useful information on perception of DDP going forward.*

3. Some aspects of the procedure are unclear: if I understand correctly, the 6 families contacted agreed to participate to the study? How were the participants in the RIGHT trial selected? In the same way, how were the participants of Adoptionplus selected?

*We agree with this comment as our recruitment procedure could be more clearly explained. DDP therapists involved in the RIGHT trial were approached to ascertain information on potential participants for this study. Families were then contacted by the research team and recruited purposively to create an adequate sample. We have described this process in the Methods section (page 8, lines 184-187) to clarify how families were identified and sampled.*

4. Do the authors, as therapists, know the participants before the study? Were they involved, and in what capacity, in patients care?

*This is an important question. No authors had any prior knowledge of the families involved in the study or were involved in patient care to any extent. A statement to clarify this has been added to the Methods section (page 10, lines 224-225)). Additionally, the co-authors, M Blair and L Tweedlie who conducted all the analysis are not therapists.*

5. The choice of IPA is insufficiently justified; it is a very specific method which does not only allow access to the lived experience of individuals (a thematic analyze also allows this). Attention to the meaning-making processes is central, and the specific contribution of this method is not well understood here.

*This comment was discussed in depth between the authors. We have agreed that during the study we held onto the individual experience of the participants throughout which is a key difference between IPA and thematic analysis. We have added a new paragraph under the heading ‘Research Design’ (page 7, lines 162-1775) to the Methods section to highlight why IPA was chosen and explain in greater detail the characteristics of IPA which make it an appropriate choice of methodology to answer our research question. Additionally, specific idiosyncrasies of participants' experience are highlighted in the results section and are given prominence in our discussion which is an important aspect of IPA research. We hope that this brings clarity to our choice of methodology.*

**RESULTS**

1. See the new terminology that Smith and his colleagues have adopted.

*Our analysis was conducted prior to the new terminology recommended by Jonathan Smith therefore we have kept the original language which is accepted by Smith.*

2. Some subthemes appear to be quite redundant. For instance, the subtheme “a very clear boundary” (p. 13) is very close to the superordinate theme of “travel”, especially with the subtheme “the driving back was decompression” (these two elements are by the way discussed together in the discussion section).

*The structure of themes and subthemes were discussed at length during this review, and there is indeed merit to the point raised. The themes highlighted above are definitely linked which is why they come after one another in the results section however we have decided not to amalgamate the themes. We believe ‘a very clear boundary’ relates to the environment the child is in during the session whereas the superordinate theme of travel, and its subthemes, are exploring the act of traveling to and from a session. We have taken time to go through our results section to update the language used to describe our subthemes and have reviewed the discussion section of these themes so that they are no longer discussed together.*

3. The part about car/public transportation journeys after the session and everything that goes on in them is really very interesting. This could be given greater prominence, particularly in the discussion section.

*Thank you for this comment, it is really appreciated. We have added more details to the Travel theme in the discussion and elaborated on the impact of car journeys after the session.*

4. Unfortunately, this study can be criticized for its lack of conceptual and methodological precision, which is precisely what is being criticized in the Smith’s methods. More precisely: what exactly is a theme (superordinate or subtheme)? What is really phenomenological in the results? Where is the interpretative account?

*In light of this comment, we focused our review on injecting more individual and meaning-making language into our results section. We have held onto the individual meaning of the DDP experience for our participant’s which is demonstrated in our results where prominence was given to those families who differed in their experience from the others.*

5. Reading the results, it's clear that this is not about the family experience of DDP, but about parents’ experience.

*Thank you for highlighting this concern. We agree that our interview questions were not clarified properly in the paper and we have elaborated on this (page 9, lines 199-202). DDP is a whole family approach, and this study explored the parents subjective view of their child’s experiences. We found that the parents were able to articulate what their child thought of a particular element, whilst also giving their own opinion. Predominantly, interview questions focussed on the parent’s perspective of their children’s experience. We do however, recognise this is a weakness of the study which we have addressed in the discussion section, and we have made suggestions for future study which includes interviews with children.*

**DISCUSSION**

1. The results are well discussed in the light of previous studies, but the discussion section does not provide any additional elements concerning the meaning-making processes regarding the therapy experience, which should nevertheless be at the heart of an IPA. I therefore suggest that the authors inject more elements regarding the processes at work in the therapeutic relationship, which they cite without detailing; for example, emotional regulation and the fact that this is a process that needs time to be worked on (link with the theme of the journey; see for instance Gross, J. J. (2001). Emotion regulation in adulthood: Timing is everything. Current directions in psychological science, 10(6), 214-219. Or: Cole, P. M., & Hollenstein, T. (Eds.). (2018). Emotion regulation: A matter of time. Routledge.). Similarly, the identification of therapist skills could be linked to empathy and the importance of intersubjectivity in child development (see Fonagy’s work).

*Thank you for recommending these interesting publications. We have taken time to read these and incorporate them into our discussion surrounding the link between travel and emotional regulation (page 29, lines 706 - 714). Fonagy’s concept of mentalisation is also included in the non-verbal communication discussion (page 31, lines 752-758) which has enriched our conversation surrounding the issue of DDP, body language, and the online delivery method. We have also related the theory of intersubjectivity to one family's individual experience of the therapeutic relationship and DDP (page 31, lines 764-772)*

2.The discussion part should be more in-depth. The current version is too short.

*We have added to the discussion section, particularly the Travel theme and Familiarity with Remote Interactions theme. We have highlighted some points below in addition to the area discussed above which has added depth to our discussion.*

- *Assumptions versus first hand experience of how different environment affects child’s engagement (p 28, lines 676 - 684)*
- *Sleep-related interventions and how this relates to children sleeping in car on journey home after therapy (page 29, lines 696 - 701)*
- *Travel time duration and access to DDP (page 30, lines 735-742)*
- *Individual family experience and fear of therapy not being effective if changed from in-person to online (page 33, lines 791-794)*

**REFERENCES**

1. Please double-check the format of the references.

*Thank you for bringing this to our attention. We have used EndNote, a trusted citation management tool to efficiently manage references which follow the Vancoucer referencing syle.*
